# Prospects for the Future

**Published:** 1894-04-28

**Authors:** 


### The Institutional Workshop.

### HOSPITAL FINANCE.

### VI.—PROSPECTS FOR THE FUTURE.

The receipts of the hospitals from sources outside those already mentioned are inconsiderable. In one or two countries the medicines prescribed to outpatients are dispensed at a small rate of profit, and drugs otherwise almost unattainable are retailed to the general public, but such a source of income cannot be reckoned either desirable or permanent.

In England and America, the nursing schools have begun to yield a definite percentage of profit to those hospitals to which they are attached. The receipts fall under two heads, viz., probationers' payments for training, and the profits on nursing institutions which supply direct from the hospitals skilled attendants to patients in their own homes from a supplementary nursing staff. The total amount which these two branches of nursing industry yielded to the hospitals in 1892, is shown by the following table:—

| | | | Probationers. | Nursing Institutions. |
|---|---|---|---|---|
| General Hospitals, | London...... | | £5,175 | £4,065 |
| ,, | ,, | Provincial | 4,331 | 6,719 |
| ,, | ,, | Scotch ... | 480 | 372 |
| ,, | ,, | Irish ... | 231 | 864 |
| Special Hospitals, | London ... | | 2,826 | 3,847 |
| ,, | ,, | Provincial | 187 | 256 |
| ,, | ,, | Scotch ... | 126 | 146 |
| ,, | ,, | Irish ... | 897 | 118 |

It is not very likely that these figures will tend to any great increase. The limit of possible probationers has been reached in most hospitals, nor will the fees bear any considerable augmentation. On the other hand, the profits on nursing institutions are more likely to show a retrograde tendency since both the managers and the public begin to feel a distaste for any large accession of income from the efforts of individual workers, often insufficiently paid.

There remains the item vaguely denominated Miscellaneous Receipts. A natural feeling of curiosity is aroused by this title, not however, to be gratified. "Sundries" in expenditure are common enough, though not altogether excusable. But that a percentage of the total income amounting in the case of Scotch hospitals to as much as 4·68 should drop as it were from the skies without apparently the possibility of mentioning how it got into the common purse, is surely a mark of some strangely muddled system of classification. The adoption of the now generally received uniform system of accounts will necessarily reduce these unsatisfactory mysteries to a minimum.

A glance at the advertising columns of any of the leading newspapers reveals only too clearly that the existing sources of hospital income are fatally deficient. Not only are enormous sums needed for extensions, alterations, and new institutions, but the every-day expenses in almost every hospital in London appear to depend as it were on a succession of miracles. If the big legacy did not fall in at the critical moment, if the unknown benefactor were not moved to a burst of liberality towards that special institution, the miserable hamper of debt must involve the work in disorganisation or even collapse. Not seldom there is a hitch in the even distribution of these saving benefactions, and though the hospital which gets more than its share is modestly silent under such a contingency, the one which comes short must struggle on with reduced staff and closed wards until the tide of affairs shall turn. The casual nature of hospital incomes cannot be better illustrated than by tracing its fluctuations according to the scale of agricultural and mercantile prosperity, according to which it is seen that precisely when the need is greatest the proportion of receipts is at its lowest ebb.

It is natural that the question of the future needs and the best methods of meeting them should, in view of these facts, press heavily on the minds of men responsible for carrying out this great work. Every year the crowd of sufferers seems to increase. The cost of maintenance has enormously increased, and will probably not again diminish. Yet no one source of hospital income has been proved to be capable of indefinite expansion. In what direction can the hospitals turn for the stable addition to their receipts which is imperatively demanded in the near future?

Any appeal to the State may be dismissed as entirely out of the question. Englishmen are justly proud of the purely voluntary character of their hospital work, and a State subsidy, involving, as it must, some proportion of State control, would be regarded as a very doubtful benefit, even if it could be obtained.

Mr. Havelock Ellis* sees a solution of the difficulty in an expansion of the rate-supported hospital or poor-law infirmary into a vast organisation which should swallow up the private practitioner and treat gratuitously rich and poor alike at the charges of a hospital rate. We have already given reasons for the conclusion that local support extorted by means of a rate is the worst of all sources to depend on for the care of the sick. So little is it likely that this delicate function should ever be placed wholly in the power of the vestries and guardians, that there is good reason to hope the time is not far distant when even the care of the aged and infirm will be removed from their jurisdiction. It would be a disastrous day for the hospitals should their administration ever be placed at the mercy of local faction, or exposed to the dangers of a reactionary retrenchment.

What, then, can be done, if recourse may be had neither to the State nor the rates? The giving power of the public can be gauged pretty accurately by the experienced. So much and no more will the springs of charity send out year by year, and this is so closely the case that a special appeal, such as that on the loss of the "Victoria," results in a perceptible lessening of gifts towards less prominent objects. Side by side with the enormous increase witnessed by the last twenty years in the charitable institutions appealing for voluntary support, is no corresponding increase in the subscribing public. Those who give most generously are forced to leave countless appeals unanswered, and the number of objects having some real claim on the purse of the average man of property is often such as to force him painfully to subdivide his donations. The rich and the generous, in fact, are about where they were, but

* "The Nationalisation of Health," by Havelock Ellis.

those to whom they minister have far outgrown their ministrations.

Yet if this be so the outlook is still full of hope. If the last twenty years have been rapid years of growth in the number of the helpless and suffering, they have witnessed also the steady growth and consolidation of, in many respects, the most hopeful democracy which the world has ever known. When the overwhelming increase of the population is carefully investigated, it will be found to be most evident in two classes, separated widely, no doubt, in ideals, but identical in interests. They may be defined as the regular wage earners at from £1 to £4 a week, and the salary earners at from £50 to £200 or £300 a year. Let the Londoner visit any of the outlying suburban districts, as unfamiliar perhaps to him as Kamschatka, and he will find mile after mile of houses inhabited indiscriminately by these two classes. He will be struck by countless indications of refinement, education, and thrift; he will see plenty of tokens also of poverty and privation. If he is permitted to know something of the lives and interests of those he visits he will discover instances of simple generosity and self-denial, which will lend him a higher ideal of giving than years of acquaintance with subscription lists and charity sermons.

It is here that the strength of the hospitals lies, and for this part of the community they in a great measure exist.

Presenting countless variations of circumstances, the members of each class defined above have this in common, that their prosperity, however substantial, is liable in the hour of illness to be turned to bitterest privation. Where in severe illness from pride or some other cause the aid of a hospital is not sought, the suffering entailed, not only on the patient, but on the whole family is incalculable, and this in cases where charitable assistance might excusably be reckoned unnecessary and undesirable. It would seem that no height of apparent prosperity can be urged as a reason for excluding those who are dependent on their own earnings from the special aid which hospitals, and they alone, can supply. And it would be hard to point out a nobler or healthier function for the hospitals than that of restoring the bread-winner, whether he wear a white jacket or black coat, to his place in the community. But let the benefit be frankly claimed, and the obligation frankly recognised. The feelings of shame on the one hand, and of distrust on the other, with which these benefits are now claimed and conceded, will be then completely effaced by the better spirit of sympathy and security which a system of mutual interdependence cannot fail to create and continue.

